# Identifying vulnerable groups in academic burnout among higher education students: lifestyle and sociodemographic characteristic

**DOI:** 10.1186/s12889-026-26486-2

**Published:** 2026-02-05

**Authors:** Marja Eliisa Holm, Jouni Lahti, Valtteri Pohjola, Suvi Parikka

**Affiliations:** Department of Public Health, The Finnish Institute for Health and Welfare, P.O. Box 30, Helsinki, 00271 Finland

**Keywords:** Academic burnout, Higher education, Latent class analysis, Lifestyle, Population-based study

## Abstract

**Background:**

Academic burnout is a major concern in higher education. We aimed to identify lifestyle groups among students using latent class analyses and to examine how sociodemographic characteristics and these lifestyle groups were associated with academic burnout.

**Methods:**

A representative population-based survey of Finnish higher education students (*n* = 3639; aged 18–34 y) was conducted in 2024. Latent class analyses of the lifestyles (physical activity, use of nicotine products, cannabis use, risky alcohol consumption, eating vegetables and fruits/berries, insufficient sleep, problematic Internet use) were used to group students. Differences in academic burnout by lifestyle groups and sociodemographic characteristics were examined using a regression analysis. Academic burnout was assessed using the School Burnout Inventory (SBI-9).

**Results:**

Female sex, disability, and financial insecurity were associated with higher academic burnout. Women with disability and financial insecurity showed the highest burnout compared to other students (B = 9.83). Four lifestyle groups were identified: healthy lifestyles (37%); physically inactive with problematic Internet use (29%); physically active with substance use (alcohol, nicotine products, cannabis; 13%); and unhealthy lifestyles (21%). Physically inactive students with problematic Internet use or unhealthy lifestyles reported higher burnout than healthy students or physically active students who used substances, with sex differences. The intersectional group—women with disabilities and financial insecurity—reported equally high levels of burnout in each lifestyle group.

**Conclusions:**

To prevent academic burnout, policies should promote healthy lifestyles among higher education students—particularly physical activity and limited screen time. Women with disabilities and financial insecurity are the most vulnerable to burnout, regardless of lifestyle; thus, preventive interventions are essential.

**Supplementary Information:**

The online version contains supplementary material available at 10.1186/s12889-026-26486-2.

## Introduction

Research interest in burnout in academic contexts has increased in recent years [1–3]. Academic burnout is characterized by a maladaptive response to long-term exposure to stressful events in educational settings among students in both universities and universities of applied sciences [2, 3]. Academic burnout encompasses three aspects: cynical attitudes toward schoolwork, exhaustion due to study demands, and feelings of inadequacy [4, 5]. Cynicism reflects decreased interest and meaning in academic duties, exhaustion refers to strain from overwork, and feelings of inadequacy indicate diminished competence and lack of accomplishment in schoolwork [4]. Academic burnout has serious consequences for students’ well-being, including increased mental health symptoms [6], school dropout [7], lower academic achievement [8], and delays in studies [9]. Therefore, understanding how lifestyles and background factors shape vulnerability to academic burnout is essential for developing preventive and equity-oriented interventions in higher education.

Most higher education entrants are young adults, typically aged 18–25. Transitioning into higher education may negatively affect lifestyle behaviors, including problematic Internet use, physical activity, diet quality, sleep, and substance use, due to increased responsibility for regulating these habits. Correlational studies indicate that problematic Internet use is associated with lower physical activity and higher substance use [10–12], while healthier dietary habits, higher physical activity, and adequate sleep are related among higher education students [13–15]. Furthermore, physical activity is positively associated with alcohol use but negatively associated with tobacco and illicit drug use [16]. Nevertheless, only a few studies have applied subgroup identification techniques, such as latent class analyses, to categorize higher education students into lifestyle groups. El Ansari et al. identified three groups—healthiest, smokers, and non-smokers with problematic drinking [17]. Nazar et al. found four groups—healthiest, healthy diet without substance use or physical activity, unhealthy diet without substance use or physical activity, and unhealthiest [18]. However, these studies did not include problematic Internet use or sleep issues. To fill this gap and address growing concerns about excessive Internet use among higher education students [19], understanding the co-occurrence of problematic Internet use and other lifestyle behaviors is essential because it provides novel knowledge and informs the development of effective interventions.

In addition, some interplay between lifestyle habits and academic burnout has been suggested. Insufficient physical activity, sleep problems, compulsive Internet use, and alcohol and illicit drug use have been associated with academic burnout among higher education students [20–26], although some studies report an insignificant association for physical activity and smoking [22, 24]. However, limited information exists on how multiple behavior patterns relate to burnout, leaving a research gap. Therefore, it is essential to examine combinations of lifestyle behaviors in relation to academic burnout, rather than considering each behavior alone. This approach may help identify distinct groups with specific lifestyle patterns most susceptible to burnout.

Sociodemographic characteristics are associated with academic burnout in higher education, although findings are mixed. Some studies have reported higher burnout in female students [24], while others have found no sex difference or higher burnout among males [27, 28]. Age appears to have no or minimal effect [1, 24], whereas disabilities and financial insecurity increase vulnerability to burnout [24, 29, 30]. However, there is a lack of evidence regarding whether the intersection of specific socioeconomic factors increases vulnerability to academic burnout. This intersectional analysis helps to understand which combinations of socioeconomic vulnerabilities most strongly shape burnout risk.

The current study bridges these knowledge gaps. The aim of this study was to identify potential distinct lifestyle groups among higher education students using a latent class analysis, based on seven lifestyle behaviors: physical activity, compulsive Internet use, healthy dietary habits, tobacco or nicotine use, cannabis use, alcohol use, and insufficient sleep. We also investigated how sociodemographic factors and lifestyle groups are associated with academic burnout. To our knowledge, no study has yet examined the relationships between distinct lifestyle groups and academic burnout using a national dataset representing the higher education student population. It was hypothesized that at least three distinct lifestyle groups would emerge: the healthiest (frequent physical activity, a healthy diet, sufficient sleep, and less frequent problematic Internet use and substance use), mixed groups with healthy and unhealthy behaviors, and the unhealthiest. Notably, there is uncertainty in these hypotheses, as prior studies did not include sleep or problematic Internet use. Additionally, membership in the unhealthiest lifestyle group was expected to be associated with the highest academic burnout. Certain sociodemographic characteristics, such as disability, were expected to increase vulnerability to academic burnout.

## Methods

### Data and design

We analyzed cross-sectional data of higher education students at universities and universities of applied sciences from the Finnish Student Health and Wellbeing Survey (KOTT) [31]. The KOTT survey is Finland’s only representative student population survey on health, well-being, academic engagement, and healthcare of higher education students. The data was collected by THL between March 18 and April 30, 2024.

The analyses are based on population representative survey data of higher education students across Finland. An invitation was e-mailed to 11,904 undergraduate students aged 18–34 years who were randomly selected from all Finnish higher education institutions using stratified random sampling. The stratification of the higher education institutions was conducted so that each sample included 140–400 students from universities of applied sciences and 150–800 from universities, totaling about 6,000 students from both sectors. Students from these institutions were chosen via random sampling, with larger institutions providing larger samples. A web questionnaire was available in Finnish, Swedish, and English. Of the invitees 3,639 (31%) participated. The survey was approved by the THL Ethics Committee (THL/5471/6.02.01/2023). Participation was voluntary and anonymous, with students giving informed consent by completing the survey.

### Measures

Appendix A presents the study questions in more detail and the dichotomization of variables, including those on academic burnout, lifestyle variables, disability, and financial uncertainty. Notably, all questions were co-developed with researchers and subject-matter experts and pilot-tested with higher education students, demonstrating good face validity.

#### Academic burnout

The School Burnout Inventory (SBI-9) was used to measure academic burnout [2, 4]. This widely used inventory has demonstrated good internal consistency and concurrent validity [2, 4]. It consists of nine items describing students’ experiences with their studies over the past month (see Appendix A). Total scores range from 0 to 45, with higher scores indicating greater burnout. In our study, the internal consistency (Cronbach’s alpha = 0.90) for the SBI-9 was excellent.

#### Lifestyle variables

Physical activity was measured using two questions: one regarding the amount and intensity of aerobic physical activity and another regarding muscle-strengthening exercise (see Appendix A). A dichotomous variable was constructed to indicate whether the respondent meets the physical activity recommendations based on the WHO’s global guidelines [32].

The use of tobacco or nicotine products was measured using two questions: one regarding the use of tobacco or nicotine products and another regarding e-cigarettes containing nicotine (see Appendix A). We report the proportion of respondents who indicated using at least one tobacco or nicotine product, at least occasionally. For cannabis, we report the proportion of respondents who used it in the past 12 months (Appendix A).

Risky alcohol consumption was evaluated using the Alcohol Use Disorder Identification Test (AUDIT-C; Appendix A). It is an effective and valid brief screening tool for problem drinking and alcohol abuse or dependence [33, 34]. A dichotomous scale was used to indicate risky alcohol consumption, with validated cutoffs of ≥ 6 for men and ≥ 5 for women (total score 0–12) [34].

The proportion of individuals with healthy dietary habits was assessed using questions about vegetable consumption and consumption of fruit or berries (see Appendix A). We focused on the proportion of respondents who reported eating vegetables or fruit/berries daily. This frequent consumption is positively associated with other dietary factors, reflecting a healthy diet and supporting construct validity [35]. For insufficient sleep, a dichotomous variable was constructed to indicate whether respondents sleep less than 7 h on weekdays, based on global recommendations for adults aged 18–64 years [36].

Problematic Internet use was assessed using a short version of the Compulsive Internet Use Scale (CIUS-5; see Appendix A) [37]. The CIUS-5 has been validated in eight languages, including Finnish, and has been shown to be a valid, reliable, and stable instrument [37]. In the present study, the internal consistency (Cronbach’s alpha = 0.80) for the CIUS-5 was good. We focused on the proportion of participants with a total score of at least 9 points (total score 0–20; higher scores indicate more severe problematic Internet use) [38, 39]. This cutoff was chosen for its high specificity in identifying at-risk participants [38].

#### Sociodemographic variables

We included the following sociodemographic variables: age group (18–26 years and 27–34 years), sex (women/men), relationship status (yes/no), higher education sector (university/university of applied sciences), disability (yes/no), and financial uncertainty (yes/no; see Appendix A). Disability was assessed using the Global Activity Limitation Indicator (GALI; see Appendix A) [40], which has been previously validated against other disability measures in several European countries [41].

### Data analysis

Survey weights were used to account for the sampling design and to reduce bias caused by non-participation. Weights were calculated using the inverse probability weighting (IPW) method [42] based on register data for the full sample, including age, sex, native language, higher education sector, and credits obtained in the previous semester. The IPW method has been applied in several survey studies and has been found to be suitable for correcting non-response rates in the Finnish population [42].

We first calculated the prevalence of sociodemographic and lifestyle variables, as well as mean academic burnout scores among higher education students. Next, a latent class analysis (LCA) was used to identify unobserved groups with similar response patterns. The LCA classified the sample into mutually exclusive latent groups based on membership probabilities [43]. The R package reglca was employed, as it allows for the incorporation of survey weights and comparison of alternative group solutions [44]. We evaluated 2–5 group models of lifestyle indicators using the Akaike Information Criterion (AIC), the Bayesian Information Criterion (BIC), the log-likelihood, and the bootstrap likelihood ratio test (BLRT). Lower AIC and BIC values indicated better fit, while higher log-likelihood values indicated an improved model fit. BLRT compares nested models, with a p-value < 0.05 indicating a superior fit for a more complex model. After selecting the optimal number of groups, item-response probabilities were examined and plotted to characterize each latent group.

We calculated the sociodemographic characteristics of lifestyle groups using Stata (Version 16), accounting for the survey design. Differences in academic burnout across sociodemographic and lifestyle groups were examined using regression analysis, controlling for other sociodemographic variables. Interaction effects between sociodemographic characteristics and lifestyle groups on burnout were also assessed. Adjusted means were estimated using the margins command [45], and unstandardized regression coefficients (*B*) were reported as measures of association.

## Results

### Characteristics of the sample

The mean age of the students was 24.65 (SD = 3.60) years, and 1,990 (54.7%) were female. Table 1 presents the prevalence of sociodemographic and healthy lifestyle variables, along with the mean level of academic burnout. Approximately 57% of students were in a relationship, almost one-fifth reported that their financial situation was very stretched and uncertain, and 31% had disability. Additionally, about 30% engaged in risky alcohol consumption, and 28% used tobacco or nicotine products. Nearly one-third had healthy dietary habits, and more than half were sufficiently physically active. Almost one-third reported problematic Internet use, and 14% reported insufficient sleep. The mean burnout score was 18.5.

**Table 1:** Sociodemographic and lifestyle characteristics and academic burnout among participants

	Higher education students
The sociodemographic variables % [95% CI]	
Sex	
Men	45.4[43.6; 47.1]
Women	54.7[52.9, 56.4]
Age group	
20–26	72.2[70.6, 73.8]
27–34	27.8[26.2, 29.4]
Higher education sector	
University	51.0[40.3, 52.8]
University of Applied sciences	49.0[47.2, 50.7]
Relationship	
No	42.8[41.1, 44.6]
Yes	57.2[55.4, 59.0]
Uncertain financial situation	
No	80.6[79.2, 82.1]
Yes	19.4[18.0, 20.8]
Disabilities	
No	69.5[67.9, 71.1]
Yes	30.5[28.9, 32.1]
Lifestyle variables % [95% CI]	
Risky alcohol consumption	30.0[28.4, 31.7]
Using tobacco or nicotine products	27.6[26.0, 29.2]
Using cannabis	10.4[9.3, 11.6]
Healthy dietary habits	31.1[29.5, 32.8]
Sufficient physical activity	54.9[54.1, 56.7]
Problematic Internet use	31.3[29.6, 32.9]
Sleep little	13.5[12.3, 14.9]
Academic burnout *M*(*SD*)	18.5(10.9)

*CI*  Confidence interval, *M*  Mean, *SD*  Standard deviation

### Lifestyle groups

LCAs were conducted for models with two to five groups (Table 2). AIC and BIC values decreased, log-likelihood values increased, and BLRT *p*-values were significant for the two- to four-group models, indicating improved fit. This trend did not continue for the five-group model, where AIC and BIC increased, log-likelihood decreased, and the BLRT *p*-value was non-significant. Accordingly, the four-group model was selected.

**Table 2:** Model-fit statistics comparisons by latent group

	AIC	log-likelihood	BIC	BLRT deviance *p*
Two groups	24474.01	-12222.01	24565.48	
Three groups	24279.44	-12116.72	24419.69	< 0.001
Four groups	24257.37	-12097.69	24446.40	0.048
Five groups	24258.23	-12098.70	24485.04	ns.

*AIC*  Akaike’s information criterion; log-likelihood, *BIC*  Bayesian information criterion, *BLRT*   Bootstrap likelihood-ratio test

**Fig. 1:**
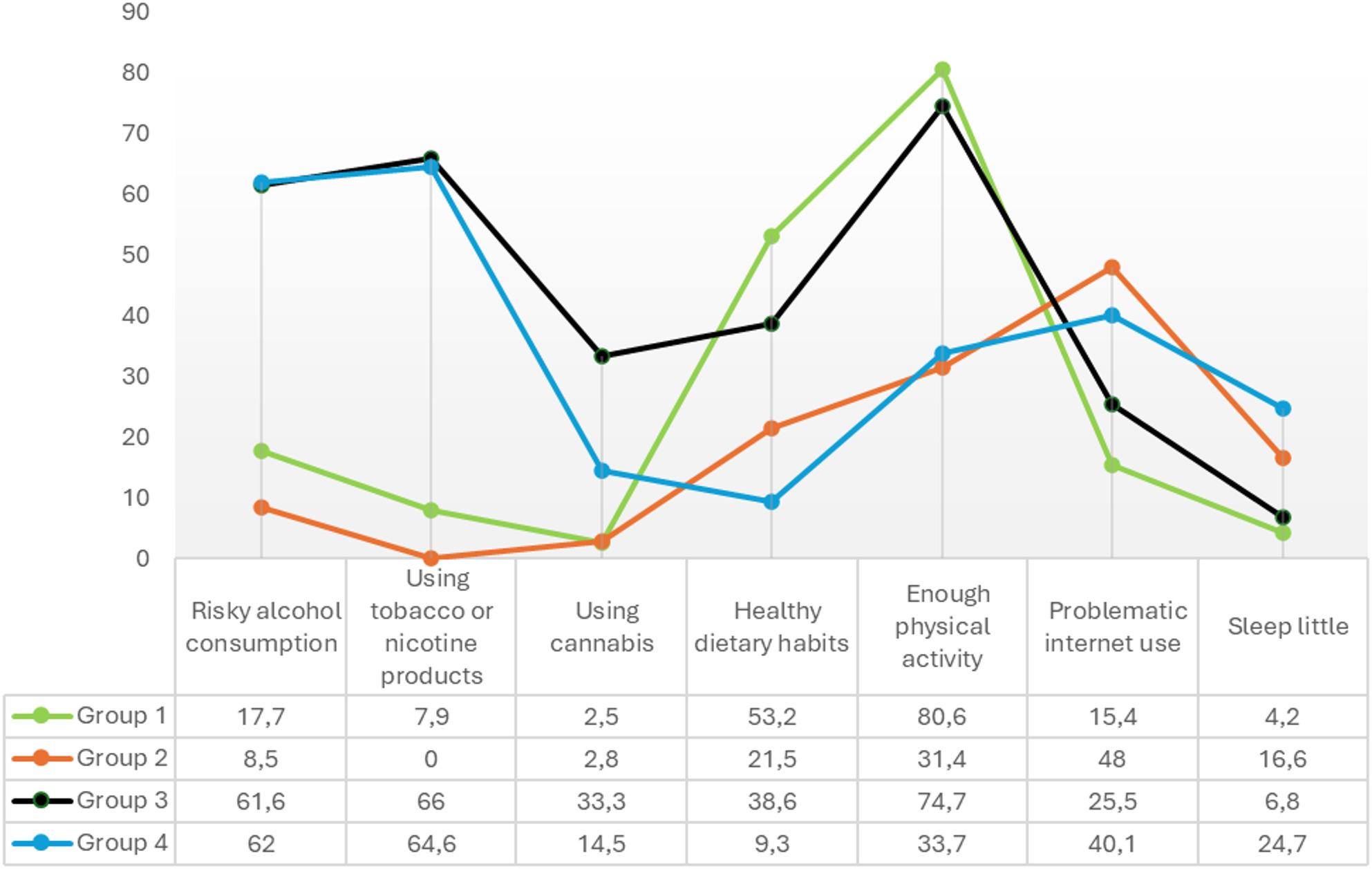
Item proportions from the latent class analysis. Group 1 (Healthy lifestyles), 37.0% *(n* = 1,285); Group 2 (Physically inactive with problematic Internet use), 29.3% (*n* = 942); Group 3 (Physically active with substance use), 12.9% (*n* = 404); Group 4 (Unhealthy lifestyles), 20.9% (*n* = 656)

Figure 1 summarizes the conditional probabilities for the healthy lifestyle groups. Approximately 37% of students belonged to the *healthy lifestyle group* (Group 1), which was characterized by a high prevalence of healthy dietary habits (53%) and physical activity (81%), along with low rates of problematic Internet use (15%), sleeping little (4%), and substance use (alcohol 18%, tobacco/nicotine 9%, cannabis 3%).

Approximately 29% of students were classified as *physically inactive with problematic Internet use* (Group 2), characterized by high rates of problematic Internet use (48%) and low rates of sufficient physical activity (31%), but low prevalence of substance use (alcohol 8%, tobacco/nicotine 0%, cannabis 3%).

Around 13% of participants belonged to the *physically active with substance use* group (Group 3), characterized by high rates of substance use (alcohol 62%, tobacco/nicotine 66%, cannabis 33%) and sufficient physical activity (75%), but a low prevalence of sleeping little (7%).

Finally, 21% of students were classified as having *unhealthy lifestyles* (Group 4), often reporting sleeping little (25%), alcohol use (62%), tobacco/nicotine use (65%), and problematic Internet use (40%), while rarely reporting healthy eating (9%) or sufficient physical activity (34%).

### Sociodemographic characteristics in lifestyle groups

Table 3 presents the prevalence of sociodemographic characteristics across lifestyle groups. There were significant differences between the groups in sex (*p*<.001), age group (*p*=.04), higher education sector (*p*<.001), financial insecurity (*p*<.001), and disability status (*p*<.001).

**Table 3:** Sociodemographic characteristics of higher education students by lifestyle groups

	Healthy lifestyles % [95% CI]	Physically inactive with problematic Internet use % [95% CI]	Physically active with substance use % [95% CI]	Unhealthy lifestyles % [95% CI]
Sex				
Men	32.9[30.1, 35.7]	26.8 [24.0, 29.5]	16.7[14.5, 18.9]	23.7 [21.1, 26.3]
Women	40.3[38.1, 42.5]^a^	31.2 [29.1, 33.3]^a^	9.8 [8.4, 11.1]^a^	18.7 [16.9, 20.5]^a^
Age group				
20–26	38.4 [36.6, 40.3]	28.0[26.5, 29.4]	12.8 [11.3, 14.2]	20.8 [19.1, 22.5]
27–34	33.2 [29.9, 36.5]^a^	32.5[29.5, 35.6]^a^	13.1[10.6, 15.5]	21.1 [18.1, 24.2]
Higher education sector				
University	42.5[40.1, 44.8]	29.2 [27.0, 31.5]	12.6 [11.0, 14.1]	15.7 [14.0, 17.5]
University of Applied sciences	31.1[28.5, 33.6]^a^	29.2 [26.7, 31.8]	13.2 [11.3, 15.2]	26.5 [24.0, 29.0]^a^
Relationship				
No	36.1[33.4, 38.8]	30.3[27.7, 33.0]	11.9[10.1, 13.8]	21.6[19.3, 24.0]
Yes	37.7[35.4, 40.0]	28.4[26.2, 30.5]	13.6[11.9, 15.3]	20.3[18.3, 22.3]
Uncertain financial situation				
No	40.5[38.5, 42.5]	28.8[26.9, 30.6]	12.0[10.7, 13.3]	18.7[17.1, 20.3]
Yes	22.3[18.9, 25.8]^a^	31.3[27.3, 35.4]	16.5[13.4, 19.7]^a^	29.8[25.8, 33.8]^a^
Disability				
No	40.8[38.6, 42.9]	26.2[24.2, 28.2]	12.6[11.1, 14.1]	20.4[18.6, 22.3]
Yes	28.5[25.6, 31.5]^a^	36.1[32.9, 39.2]^a^	13.5[11.3, 15.8]	21.9[19.1, 24.6]

*CI *Confidence interval. ^a^Significant differences in sociodemographic characteristics between lifestyle groups (*p* < .05)

*The healthy lifestyle group* (Group 1) included more women than men, more younger students (aged 20–26) than older students (aged 27–34), more university students than students from universities of applied sciences, more students without financial insecurity than those with it, and more students without disability than those with disability. *The physically inactive group with problematic Internet use* (Group 2) consisted of more women than men, more older students (aged 27–34) than younger students (aged 20–26), and more individuals with disabilities than without. *The physically active group with substance use* (Group 3) comprised more men than women and more students reporting financial insecurity than those without it. *The unhealthy lifestyles group* (Group 4) included more men than women, more students from universities of applied sciences than from universities, and more students with financial insecurity than those without it.

### Academic burnout

**Fig. 2:**
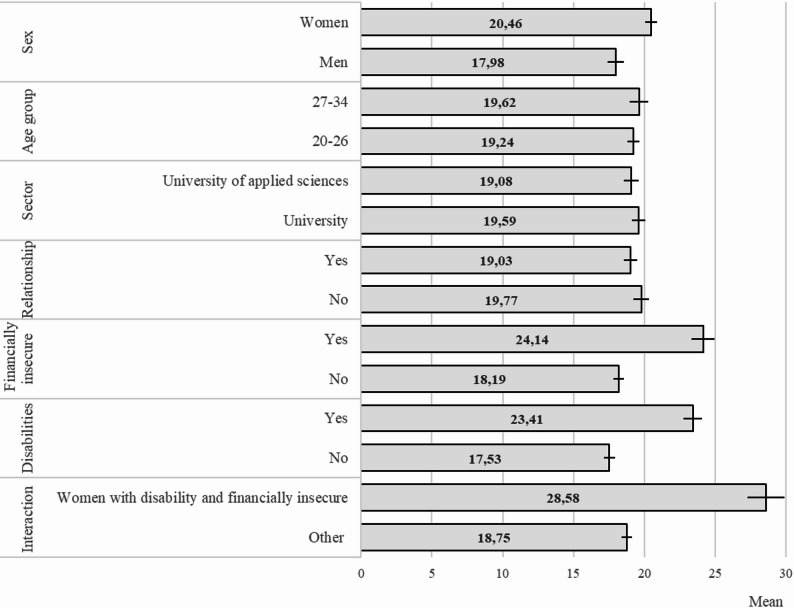
Academic burnout among higher education students by sociodemographic variables, with 95% confidence intervals. Adjusted for other sociodemographic covariates

Students with financial insecurity (B = 5.94), disabilities (B = 5.88), and women (B = 2.48) had higher academic burnout scores than their counterparts—students without financial insecurity, without disabilities, and men (Fig. 2; Table 4). The relationship status, higher education sector, and age group were not significantly associated with burnout. A significant three-way interaction—financial situation × disability × sex (*p*<.001)—was found for burnout. Women with disabilities who were financially insecure exhibited the highest levels of academic burnout (B = 9.83; *M* = 28.58) compared to other students (*M* = 18.75).

**Table 4:** Differences in academic burnout by sociodemographic features

	M [95% CI]	B [95% CI]
Sex		
Men	17.98[17.40, 18.55]	ref.
Women	20.46[20.04, 20.87]	2.48 [1.76, 3.20]***
Age group		
20–26	19.24[18.82, 19.64]	ref
27–34	19.62[18.97, 20.27]	ns.
Higher education sector		
University	19.59[19.14, 20.04]	ref.
University of applied sciences	19.08[18.56, 19.59]	ns.
Relationship		
No	19.77[19.24, 20.29]	ref.
Yes	19.03[18.57, 19.48]	ns.
Financially insecure		
No	18.19 [17.82, 18.58]	ref.
Yes	24.13 [23.32, 24.94]	5.94 [5.02, 6.85]***
Disability		
No	17.53[17.13, 18.93]	ref.
Yes	23.41[22.75, 24.08]	5.88 [5.09, 6.67]***
Significant interaction (Women x disability x financially insecure)		
Other	18.75[18.38, 19.13]	ref.
Women with disability and financially insecure	28.58 [27.28, 29.89]	9.83[8.47, 11.19]***

Adjusted for other sociodemographic covariates

*M *Mean, *CI *Confidence interval, *B *Standardized regression coefficient, *ref.* Reference group, *ns.* Insignificant

*p < .05; **p < .01; ***p < .001

**Fig. 3:**
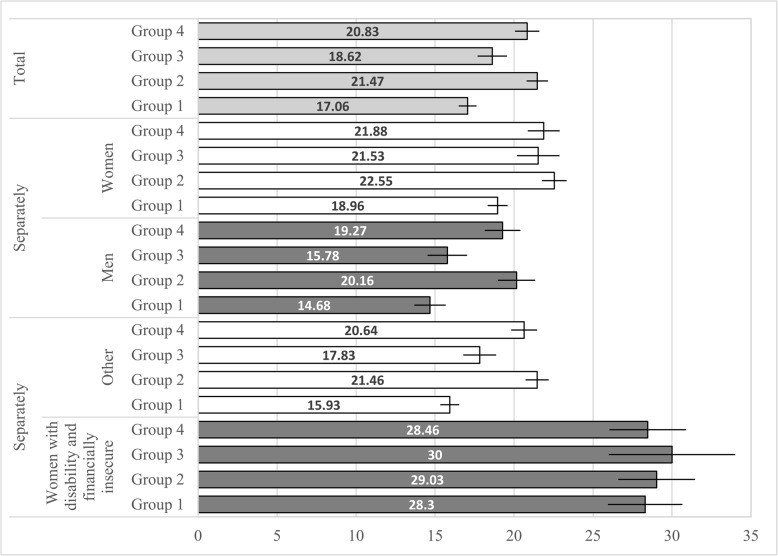
Academic burnout among higher education students, with 95% confidence intervals, by lifestyle groups. Healthy lifestyles (Group 1), physically inactive with problematic Internet use (Group 2), physically active with substance use (Group 3), and Unhealthy lifestyles (Group 4); adjusted for sociodemographic covariates

Students who were physically inactive with problematic Internet use (Group 2), or who had unhealthy lifestyles (Group 4) reported higher academic burnout (*p*<.001) than students in the other groups: healthy lifestyles (Group 1) and physically active with substance use (Group 3; see Fig. 3; Table 5). Students who were physically active and used substances reported slightly higher burnout (*p*<.01) than healthy lifestyle students.

**Table 5:** Differences in academic burnout between lifestyle groups

	M [95% CI]	B [95% CI]
Total		
Group 1. Healthy lifestyles	17.06 [16.50, 17.62]	ref.
Group 2. Physically inactive with problematic Internet use	21.47 [20.79, 22.14]	4.41 [3.53, 5.28]***
Group 3. Physically inactive with substance use	18.62 [17.69, 19.54]	1.56 [0.47, 2.65]**
Group 4. Unhealthy lifestyles	20.83 [20.06, 21.60]	3.77 [2.81, 4.73]***
Group 3 (ref.) vs. Group 2		2.85[1.69, 4.00]***
Group 4 (ref.) vs. Group 2		ns.
Group 3 (ref.) vs. Group 4		2.21[1.00, 3.42]***

	**M [95% CI]**	**B [95% CI]**
Men		
Group 1. Healthy lifestyles	14.68 [13.69, 15.66]	ref.
Group 2. Physically inactive with problematic Internet use	20.16 [18.98, 21.34]	5.49[3.91, 7.05]***
Group 3. Physically active with substance use	15.78 [14.53, 17.03]	ns.
Group 4. Unhealthy lifestyles	19.27 [18.14, 20.39]	4.59[3.07, 6.10]***
Group 3 (ref.) vs. Group 2		4.38[2.65, 6.11]***
Group 4 (ref.) vs. Group 2		ns.
Group 3 (ref.) vs. Group 4		3.49[1.80, 5.17]***
Women		
Group 1. Healthy lifestyles	18.96[18.33, 19.6]	ref.
Group 2. Physically inactive with problematic Internet use	22.55[21.77, 23.34]	3.59[2.57, 4.60]***
Group 3. Physically active with substance use	21.53[20.18, 22.89]	2.57[1.07, 4.06]**
Group 4. Unhealthy lifestyles	21.88[20.87, 22.90]	2.92[1.70, 4.14]***
Group 3 (ref.) vs. Group 2		ns.
Group 4 (ref.) vs. Group 2		ns.
Group 3 (ref.) vs. Group 4		ns.

	**M [95% CI]**	**B [95% CI]**
Women with disability and financially insecure		
Group 1. Healthy lifestyles	28.30[25.94, 30.65]	ref.
Group 2. Physically inactive with problematic Internet use	29.03[26.60, 31.46]	ns.
Group 3. Physically active with substance use	30.00[26.00, 34.00]	ns.
Group 4. Unhealthy lifestyles	28.46[26.04, 30.88]	ns.
Group 3 (ref.) vs. Group 2		ns.
Group 4 (ref.) vs. Group 2		ns.
Group 3 (ref.) vs. Group 4		ns.
Other		
Group 1. Healthy lifestyles	15.93 [15.33, 16.53]	ref.
Group 2. Physically inactive with problematic Internet use	21.46 [20.73, 22.19]	5.53 [4.59, 6.48]***
Group 3. Physically active with substance use	17.83 [16.79, 18.88]	1.91 [0.71, 3.11]***
Group 4. Unhealthy lifestyles	20.64 [19.82, 21.47]	4.72 [3.69, 5.75]***
Group 3 (ref.) vs. Group 2		3.63[2.35, 4.90]***
Group 4 (ref.) vs. Group 2		ns.
Group 3 (ref.) vs. Group 4		2.81[1.48, 4.14]***

Adjusted for other sociodemographic covariates

*M *Mean, *CI *Confidence interval, *B *Standardized regression coefficient, *ref.* Reference group, *ns.* Insignificant

*p < .05; **p < .01; ***p < .001

There was a significant interaction between sex and lifestyle group on academic burnout (*p*<.001). Among women, all three unhealthy lifestyle groups—physically inactive with problematic Internet use (B = 3.59), physically active with substance use (B = 2.57), and unhealthy lifestyles (B = 2.92)—had higher burnout than the healthy lifestyle group. Among men, only the physically inactive with problematic Internet use (B = 5.49) and unhealthy lifestyle groups (B = 4.59) showed higher burnout than the healthy lifestyle group; those who were physically active with substance use did not.

We found a significant interaction (*p*<.001) between lifestyle groups and the most vulnerable group—women with disabilities and financial insecurity—on academic burnout. Students who were simultaneously women, disabled, and financially insecure reported equally high levels of burnout regardless of their lifestyle group. Among other students, burnout was highest in the physically inactive/problematic Internet user group and the unhealthy lifestyles group.

## Discussion

Our main findings identified four distinct lifestyle groups among higher education students: healthy lifestyles (37%), physically inactive with problematic Internet use (29%), physically active with substance use (13%), and unhealthy lifestyles (21%). Students who were physically inactive with problematic Internet use or who had unhealthy lifestyles were more prone to academic burnout than other groups, with differences observed between sexes. Additionally, women with disabilities and financial insecurity exhibited the highest levels of academic burnout compared with other students, and they reported equally high burnout regardless of whether they belonged to healthy or unhealthy lifestyle groups.

### Lifestyle groups among higher education students

Our identification of the healthy lifestyle group highlighted that over one-third of higher education students engage in multiple health-promoting behaviors simultaneously. This finding corroborates earlier evidence that positive health behaviors—such as a healthy diet, regular physical activity, and rare substance use—characterize a subgroup of higher education students [18]. We further found that these students often reported adequate sleep, while nearly one in six still experienced problematic Internet use. Thus, even among healthy students, certain unhealthy behaviors may occur. Our study also showed that nearly one-third of higher education students belonged to the group characterized by physical inactivity and problematic Internet use, but low engagement in substance use. Previous research has linked problematic Internet use to reduced physical activity and increased substance use [10–12]. Our results extend this understanding by showing that problematic Internet use and physical inactivity can co-occur independently of substance use, highlighting the heterogeneity of lifestyles among individual groups. These behaviors may co-occur because problematic Internet use can displace time for physical activity [11], or because physically inactive individuals may miss out on exercise-related physiological benefits that help regulate addictive and problematic Internet use behaviors [10].

Our findings highlight the coexistence of physical activity and substance use (alcohol, tobacco/nicotine, and cannabis use) among the group of higher education students. While previous research has linked physical activity to higher alcohol use but lower tobacco and other drug use [16], our results further indicate that high physical activity and substance use can occur simultaneously. Both may activate similar reward pathways in the brain and reduce anxiety and stress, leading some students to engage in both to prolong positive experiences, potentially explaining their co-occurrence [46]. We also identified an unhealthy lifestyle group, extending previous work that characterized such groups by poor diet, low physical activity, and high substance use [18]. In our study, this group also reported often sleeping little and experiencing problematic Internet use, suggesting that unhealthy habits may reinforce one another, creating self-perpetuating cycles that are challenging to address.

Our findings indicate that the sociodemographic characteristics varied across lifestyle groups. We found that the healthy group included students who were younger, female, without disabilities, financially secure, and studying at universities, suggesting that students with these characteristics may possess greater resources, motivation, and resilience, enabling them to maintain healthier lifestyle behaviors. In contrast, women, older students, and students with disabilities were overrepresented in the physically inactive group with problematic Internet use, while men, students from universities of applied sciences, or financially insecure students were more common in the physically active group with substance use or in the unhealthy lifestyle group. These results indicate that sociodemographic factors can increase vulnerability to unhealthy lifestyles and suggest that interventions may benefit from being tailored to specific student subgroups.

### Academic burnout across lifestyle and sociodemographic groups

We found that physically inactive students with problematic Internet use or unhealthy lifestyles reported the highest levels of academic burnout. In contrast, students with healthy lifestyles showed the lowest burnout levels, and physically active students with substance use showed only slightly higher burnout compared to those with healthy lifestyles. Previous research has identified associations between individual lifestyle behaviors and academic burnout [20, 22, 24, 47]. Our study extends this evidence by revealing distinct lifestyle patterns among students who are particularly vulnerable to burnout.

Our results showed that financial insecurity, disability, and being female were associated with higher levels of academic burnout among higher education students, consistent with previous evidence linking these factors to elevated burnout risk [2, 24, 29, 30]. In contrast, age, relationship status, and higher education sector (university vs. university of applied sciences) were not significantly associated with burnout, aligning with prior findings regarding age [24] and extending this evidence to the relationship status and educational sector. To date, our study is the first to demonstrate that the intersection of being a woman, having disability, and experiencing financial insecurity heightens the vulnerability to academic burnout. Female students facing both disability and financial insecurity may be particularly vulnerable to burnout due to lower self-esteem, reduced resilience to adversity, negative peer attitudes, and insufficient social or institutional support [48–51].

However, our interaction analyses revealed that the association between lifestyle patterns and academic burnout differed only by sex, with no interactions observed for other sociodemographic factors. This indicates that, apart from sex, lifestyle-related risk and sociodemographic vulnerabilities generally contribute independently, rather than interactively, to academic burnout. Physically inactive men with problematic Internet use or unhealthy lifestyles reported higher levels of academic burnout compared to those with healthy lifestyles, whereas physically active men with substance use did not differ in terms of burnout. Among women, all three unhealthy lifestyle groups exhibited higher levels of burnout than the healthy lifestyle group. Physically inactive women and men with problematic Internet use may be particularly susceptible to burnout, as they may miss out on the stress-relieving benefits of physical activity and may experience challenges with concentration or a sense of meaninglessness in studying due to excessive Internet use [47, 52, 53]. Increased academic burnout may lead to students becoming too fatigued to engage in physical activity or to use the Internet as a coping mechanism [47, 52], thereby perpetuating a negative cycle. Among students with unhealthy lifestyles, physical inactivity combined with problematic Internet use may also contribute to academic burnout. Inadequate sleep may further impair concentration and emotional regulation [54], while poor nutrition, alcohol, and substance use may negatively affect cognitive processes and learning [55, 56], all of which may exacerbate burnout. However, we found no significant differences in burnout between students with unhealthy lifestyles and those with only physical inactivity and problematic Internet use behaviors, suggesting that physical inactivity and problematic Internet use may be the primary contributors to burnout in higher education.

Our study indicated that only physically active women with substance use, but not men, were prone to academic burnout. Substance use can impair concentration and learning performance [55], leading to burnout. This can become a vicious cycle, where students begin using substances to cope with their burnout [26]. Our finding suggests that women may be more vulnerable to this cycle, with physical activity not protecting them from burnout. In contrast, for men, regular physical activity may help prevent burnout despite substance use.

Moreover, we found that the vulnerable group—women with disabilities and financial insecurity—reported equally high levels of academic burnout in both healthy and unhealthy lifestyle groups. By contrast, among other students, unhealthy lifestyle behaviors were associated with higher burnout. These findings suggest that the vulnerability to burnout associated with these intersecting disadvantages may not be mitigated through healthy lifestyle choices, highlighting that lifestyle-related and sociodemographic vulnerabilities contribute independently, rather than interactively, to burnout.

### Limitations and strengths

A key strength of this study is the use of nationally representative survey data of higher education students in Finland, encompassing a broad range of sociodemographic and lifestyle factors. Although the response rate was low, weighting based on register data corrected for nonparticipation bias, enhancing generalizability. Academic burnout was assessed using a validated, internationally recognized instrument [2, 4]. Taken together, the study provides novel evidence on how different lifestyle patterns are associated with academic burnout in a nationally representative higher education population.

The present study also has some limitations. First, the cross-sectional design prevents any causal inferences regarding lifestyle behaviors, sociodemographic characteristics, and academic burnout. Second, it relied on self-report measures, which may increase the likelihood of social desirability bias. Third, the study had a relatively low response rate (31%), which may limit the representativeness of the sample despite the use of weighting procedures to adjust for demographic differences. Fourth, lifestyle variables were dichotomized. This approach was applied to identify participants at higher risk for problematic behaviors (e.g., risky alcohol use, problematic Internet use), allowing for clearer interpretation. However, dichotomization may reduce variability and obscure more subtle behavioral patterns, which can limit the nuanced interpretation of lifestyle effects. Finally, the study included only higher education students in Finland; thus, replication in other cultural contexts is needed to enhance international generalizability.

Our study focused on the effects of healthy lifestyles and sociodemographic factors, which were assessed comprehensively. Future longitudinal and experimental research is needed to deepen understanding of the underlying mechanisms linking lifestyle behaviors and sociodemographic characteristics to academic burnout. Moreover, not all potential factors related to academic burnout (e.g., learning pressure, school climate, lack of support, learning-related self-esteem, and concerns about academic achievement) were examined [5, 50, 57]. Future research would also benefit from including measures of externalizing problems [58], as they may be closely linked to lifestyle risk behaviors and academic burnout.

## Conclusion

To conclude, our study offers three novel contributions to the existing literature: (i) the co-occurrence of physical inactivity and problematic Internet use plays a significant role in academic burnout; (ii) clear sex differences are observed in the relationship between lifestyle and burnout; and (iii) women with disabilities and financial insecurity are vulnerable to burnout, regardless of lifestyle.

Our results showed four distinct groups (healthy lifestyles; physically inactive with problematic Internet use; physically active with substance use; and unhealthy lifestyles). Both physically inactive students with problematic Internet use and those with unhealthy lifestyles reported the highest levels of academic burnout. To prevent academic burnout, policies should particularly promote physical activity and prosocial behaviors to limit excessive Internet use among higher education students [59]. A web-based social program that develops self-regulation skills—like goal setting, monitoring, and self-assessment—can help prevent Internet addiction and improve self-regulation in higher education students [60]. These interventions may be particularly beneficial among men, as we found that low physical activity combined with problematic Internet use was associated with their academic burnout. In turn, women showed high burnout in all unhealthy lifestyle groups, highlighting the need to develop comprehensive approaches addressing multiple lifestyle factors to prevent academic burnout. Finland’s national nutrition policies, including student meal subsidies, play an important role in ensuring vulnerable students have at least one healthy, affordable meal per day. Students should also be encouraged to use affordable physical activity services offered by universities and student organizations.

Our study also highlighted that the intersection of being female, having disability, and experiencing financial insecurity increases vulnerability to burnout, regardless of whether they follow healthy or unhealthy lifestyles. Instead of focusing largely on lifestyle promotion campaigns, the results imply at the policy level that it may be more efficient to prevent academic burnout through public interventions. Student health services need sufficient resources to prevent their burnout, and student social security should not be weakened, as vulnerable groups would be particularly affected by these cuts. Targeted interventions could be implemented as part of the higher education system, including acceptance and commitment training, which fosters mindfulness and psychological flexibility [61], as well as social support networks involving teachers and professionals, to help prevent academic burnout among vulnerable students.

## Supplementary Information

Supplementary Material 1.

## Data Availability

The datasets analyzed during the current study are not publicly available because the data processing regulations of the Finnish Institute for Health and Welfare (THL) require an approved data permit before any data can be shared. Researchers may request access for scientific purposes through the Social and Health Data Permit Authority (Findata) by submitting a research proposal along with an approved user authorization application.
