# Supplementary material for: Identifying vulnerable groups in academic burnout among higher education students: lifestyle and sociodemographic characteristic
Source: BMC Public Health. 2026 Feb 5;26:826. doi: 10.1186/s12889-026-26486-2 (PMC12973672; doi:10.1186/s12889-026-26486-2)
Supplement: Supplementary file 1 — Supplementary Material 1. [file 12889_2026_26486_MOESM1_ESM.docx]

**Appendix A.** Used study questions

| **Academic burnout** 1) I feel like I’m drowning under the amount of work associated with my studies; 2) I feel apathetic about my studies and often think about dropping out; 3) I often feel inadequate as a student; 4) I often sleep poorly due to my studies weighing on my mind; 5) I feel like I’m losing interest in my studies; 6) I often wonder if my studies matter at all; 7) I worry about my studies even in my spare time; 8) I used to have higher expectations for myself in regard to my studies; and 9) The pressure of my studies is causing trouble in my relationships. *Response options:* a) Strongly disagree, b) Disagree, c) Partially disagree, d) Partially agree, e) Agree, f) Strongly agree. |
| --- |
| **Physical activity** 1) How much exercise do you get in a week in the course of your studies, work, commute, and spare time during the past 12 months? *Sub-options:* a) hardly any regular weekly exercise; b) low-intensity aerobic exercise (does not make you sweat or run out of breath, e.g., walking leisurely); c) moderate-intensity aerobic exercise (makes you sweat a bit and/or get slightly out of breath, e.g., walking briskly); d) high-intensity aerobic exercise (makes you sweat a lot and/or run out of breath, e.g., jogging or running). Respondents reported hours and minutes of weekly exercise.  2) On how many days during an ordinary week do you engage in exercise that maintains or develops your muscle strength? Examples include exercising at a gym, home exercises, fitness classes, ball games and racket sports, or physically strenuous household chores.  We report the proportion of participants who engage in at least 2 hours and 30 minutes of moderate-intensity aerobic activity, or 1 hour and 15 minutes of vigorous-intensity aerobic activity per week, or an equivalent combination of moderate- and vigorous-intensity activity, as well as engaging in muscle-strengthening activities at least twice a week. |
| **Tobacco or nicotine products** I) Do you use or have you previously used the following tobacco or nicotine products? 1) cigarettes, 2) snuff, 3) nicotine pouches, 4) heated tobacco products. *Response options:* a) not at all; b) I have previously, but I have quit; c) yes, less often than once a week; d) yes, weekly, but not every day; e) yes, daily.  II) Do you use e-cigarettes that contain nicotine? *Response options:* a) not at all; b) I have tried it; c) yes, occasionally; d) yes, daily.  We report the proportion of respondents who indicated using at least one type of tobacco or nicotine product (responses: yes, less often than once a week; yes, weekly, but not every day; or yes, daily) or using nicotine-containing e-cigarettes (responses: yes, occasionally; or yes, daily). |
| **Cannabis** Have you used cannabis at least once in the last 12 months?  *Response options*: a) no, b) yes.  We report the proportion of respondents who answered “yes” to having used cannabis. |
| **Alcohol questions (AUDIT-C)** 1) How often do you drink beer, wine, or other alcoholic beverages? Also include the times when you only had a small amount, e.g., a bottle of medium-strength beer or a sip of wine. *Response options:* a) never, b) around once a month or less, c) 2–4 times a month, d) 2–3 times a week, e) 4 or more times a week.  2) How many drinks containing alcohol do you have on a typical day when you are drinking? *Response options:* a) 1–2 servings, b) 3–4 servings, c) 5–6 servings, d) 7–9 servings, e) 10 or more servings.  3) How often have you had six or more drinks on one occasion? *Response options:* a) never, b) less than once a month, c) once a month, d) once a week, e) daily or almost daily.  Each question was scored from 0 to 4, resulting in a total score ranging from 0 to 12. A dichotomous scale was used to indicate risky alcohol consumption, with cutoffs of ≥6 for men and ≥5 for women. |
| **Dietary habits**  1) How often have you eaten vegetables (not including potatoes) in the last 7 days? 2) How often have you eaten fruit or berries in the last 7 days? *Response options:* a) not at all, b) 1–2 days, c) 3–5 days, d) 6–7 days, e) several times a day.  We focused on the proportion of respondents who reported eating vegetables or fruit/berries several times a day, or both vegetables and fruit/berries 6–7 days per week. |
| **Sleeping**  1) What time do you usually go to bed on study days/workdays?  2) What time do you usually wake up on study days/workdays?  Note: The length of nighttime sleep was calculated from the time of going to bed to the time of waking up.  The proportion of those sleeping little included respondents who slept fewer than 7 hours on weekdays. |
| **Problematic Internet use** 1) Do you find it difficult to stop using the Internet? 2) Do others (e.g., partner, friends, parents, or children) say you should use the Internet less? 3) Are you short of sleep because of the Internet? 4) Do you neglect your daily obligations (school, work, or family life) because you prefer to go on the Internet? 5) Do you go on the Internet when you are feeling down? *Responses:* rated from 0 (‘never’) to 4 (‘very often’).  Total scores ranging from 0 to 20. Higher scores indicate more severe compulsive Internet use. We focused on the proportion of participants with a total score of at least 9 points. |

| **Financial uncertainty**  How well have you managed financially in the last 12 months? *Response options:* a) very well, b) well, I have managed by living frugally, c) I have felt stretched and unsure financially. Focused on the proportion of those who answered c. |
| --- |
| **Disability (GALI)**  Consists of two questions: 1) Are you limited because of a health problem in activities that people usually do? *Response options:* a) severely limited, b) limited but not severely, c) not limited at all.  2) Have you been limited for at least the past six months? *Response options:* a) yes, b) no.  Those who responded that they had been limited but not severely or severely limited for at least the previous six months were categorized as having disabilities; others were categorized as having no disabilities |
